# Screening the genome for HCC-specific CpG methylation signatures as biomarkers for diagnosis and prognosis evaluation

**DOI:** 10.1186/s12920-021-01015-9

**Published:** 2021-06-19

**Authors:** Rui-kun Zhang, Jia-lin Liu

**Affiliations:** 1 Health Science Center, Shenzhen University, Shenzhen, China; 2 Department of Hepatobiliary and Pancreatic Surgery, Shenzhen Traditional Chinese Medicine Hospital, No.1 Fuhua Road, Shenzhen, 518000 Guangdong China

## Abstract

**Background:**

Hepatocellular carcinoma (HCC) is one of the most common and invasive malignant tumors in the world. The change in DNA methylation is a key event in HCC.

**Methods:**

Methylation datasets for HCC and 17 other types of cancer were downloaded from The Cancer Genome Atlas (TCGA). The CpG sites with large differences in methylation between tumor tissues and paracancerous tissues were identified. We used the HCC methylation dataset downloaded from the TCGA as the training set and removed the overlapping sites among all cancer datasets to ensure that only CpG sites specific to HCC remained. Logistic regression analysis was performed to select specific biomarkers that can be used to diagnose HCC, and two datasets—GSE157341 and GSE54503—downloaded from GEO as validation sets were used to validate our model. We also used a Cox regression model to select CpG sites related to patient prognosis.

**Results:**

We identified 6 HCC-specific methylated CpG sites as biomarkers for HCC diagnosis. In the training set, the area under the receiver operating characteristic (ROC) curve (AUC) for the model containing all these sites was 0.971. The AUCs were 0.8802 and 0.9711 for the two validation sets from the GEO database. In addition, 3 other CpG sites were analyzed and used to create a risk scoring model for patient prognosis and survival prediction.

**Conclusions:**

Through the analysis of HCC methylation datasets from the TCGA and Gene Expression Omnibus (GEO) databases, potential biomarkers for HCC diagnosis and prognosis evaluation were ascertained.

**Supplementary Information:**

The online version contains supplementary material available at 10.1186/s12920-021-01015-9.

## Background

Hepatocellular carcinoma (HCC) is one of the most common and invasive malignant tumors in the world. It is the most common primary malignant tumor of the liver and the third most common cause of cancer-related death worldwide [1–3]. In China, liver cancer is the fourth most common type of cancer and the third most common cause of cancer-related death [4]. The incidence rate of HCC is increasing steadily [5]. According to the Surveillance, Epidemiology, and End Results (SEER) program, the HCC incidence rate increased by 3.1% annually from 2008 to 2012. The incidence rates for males and females were 11.5 and 3.9 per 100,000. Likewise, the mortality rates for males and females with HCC increased by 2.8% and 3.4%, respectively, occupying the top spot for the deadliest cancer during the period [6]. In 2012 alone, there were 782,000 new HCC cases and 745,000 HCC-related deaths, accounting for 9.1% of all cancer fatalities. It is estimated that by 2025, more than a million cases of HCC will be diagnosed annually [7, 8].

According to estimations, more than 50% of newly diagnosed HCC cases occur in China [8]. More than half of HCC-related fatalities occur in China [9]. The primary risk factors for HCC include hepatitis B virus (HBV) infection, hepatitis C virus (HCV) infection, non-alcohol-related steatohepatitis, exposure to aflatoxin and chronic alcohol poisoning [10, 11]. HBV and HCV infection are the primary reasons for chronic liver disease and HCC in China. The incidence rates of HCV-related chronic liver cancer and HCC are also increasing [12, 13].

Most HCC patients do not display significant symptoms in early disease stages, so they are often diagnosed after the disease reaches stage III or IV. Liver resection, liver transplantation, radiofrequency ablation, transarterial chemoembolization and sorafenib are the primary clinical treatments for HCC [14]. While progress has been made in HCC clinical treatment and medical management, since most patients undergoing these treatments have late-stage cancer, the overall prognosis for HCC patients remains unsatisfactory, with a 17% 5-year survival rate [15, 16].

Alpha-fetoprotein (AFP) is a commonly used cancer marker for early screening, diagnosis and treatment evaluation. However, due to its limited sensitivity and specificity, this serum biomarker is of limited usefulness in the treatment of early-stage cancer patients [17–20]. Therefore, the American Association for the Study of Liver Diseases (AASLD) and European Association for the Study of the Liver (EASL) do not mandate using AFP for HCC diagnosis. Thus, finding a better biomarker for HCC diagnosis is an extremely important research direction.

Changes in DNA methylation are important epigenetic events for cancer. The term “epigenetic” refers to changes in gene expression mediated by mechanisms other than alterations in the primary nucleotide sequence of a gene [21, 22]. DNA methylation refers to reactions using S-adenosyl-methionine as a methyl donor that are catalyzed by enzymes called DNA methyltransferases (DNMTs), which add methyl groups to the cytosine ring to form methyl cytosines [23]. In mammalian gene sets, methylation only occurs at the 5’ position of cytosines adjacent to guanines in CpG sites [21]. CpG islands in the gene promoter regions are the most common sites. High levels of methylation at these promoter regions represent the most evident epigenetic changes. Every type of human tumor is related to the methylation of genes [24, 25]. A high level of methylation in the CpG island in the promoter region stops the combination of DNA polymerase and transcription factors. Thus, the transcription of the target gene is inhibited [26]. For example, DNA methylation can deactivate tumor suppressor genes and DNA repair genes by reducing its transcriptional activity and can reduce the expression of E-cadherin. These effects allow the development of tumors [27]. Manel Esteller et al. [28] summarized the relationship between gene methylation and various types of cancer, such as bladder cancer, cervical cancer, melanoma and glioma, with p16 methylation as a trait; high levels of p14 and APC methylation are common in gastrointestinal cancer (such as colon cancer and stomach cancer), while GSTP1 demonstrates high methylation levels in breast cancer and prostate cancer.

In HCC, O^6^-methylguanine-DNA-methyltransferase (MGMT) is another important DNA repair gene, and it is most active in the liver. The methylation of promoter region CpG islands reduces or eliminates MGMT expression in HCC cases [29, 30]. A total of 67.86% of HCC cases exhibit a high level of p73 gene methylation. The inactivation of p16INK4a caused by the methylation of promoters is also one of the main causes of HCC [27].

The methylation of DNA can be detected in phlegm, bronchial irrigation fluid, urine, blood, catheter fluid, lymph nodes and cancer tissues [22]. Thus, DNA methylation has great potential as a biomarker for the early diagnosis of cancer. A high level of SEPT9 gene methylation can be used to diagnose colorectal cancer [31]. A prognostic model of 7 CpG sites has already been applied in oral squamous cell carcinoma (OSCC) [32]. There have been reports of six methylation biomarkers that can be used to differentiate HCC patients and healthy humans [33]. Xu et al. [34] discovered 10 types of methylation biomarkers that can be used to diagnose HCC, but previous research lacked specificity. These biomarkers cannot be used to completely differentiate HCC and other types of cancer. In addition, the causal relationship between DNA methylation status and HCC outcome is still not clear. Thus, we integrated The Cancer Genome Atlas (TCGA) and Gene Expression Omnibus (GEO) Illumina 450 K DNA methylation datasets for HCC for this research to identify potential biomarkers for HCC diagnosis and prognosis.

### Methods

## Data preparation

We downloaded data for 377 cases (with paired tumor and paracancerous tissues) of HCC. The data included methylation data based on Illumina Human Methylation 450 BeadChip’s tertiary methylation datasets, genetic expression data and relevant clinical data from https://gdc-portal.nci.nih.gov/. The paired tumor and paracancerous tissues were used to analyze different methylation sites. These data were collectively used as the training set.

Two DNA methylation datasets (GSE157341 and GSE54503) and the relevant clinical data were downloaded as the validation set. We downloaded methylation data for 17 other cancers from the following TCGA datasets: BLCA, BRCA, CESC, CHOL, COAD, ESCA, KIRC, KIRP, LUAD, LUSC, PAAD, PCPG, PRAD, READ, SKCM, THCA and UCEC. The methylation datasets for 15 other types of cancer were not chosen due to the lack of paired paracancerous tissue samples. The beta (β) value of the methylation level is expressed as M/(M + U + 100). M is the number of Infinium II probes (methylated), while U is the number of Infinium I probes (nonmethylated).

## Data preprocessing

R (v 4.0.3) was used to import the various methylation datasets, genetic data, and clinical data. The Impute package was used to fill in the missing values for methylation data. The Champ package [35] and minfi package [36] were used to filter (probes with detection *p* values > 0.01, probes with < 3 beads in at least 5% of samples per probe, non-CpG probes contained in the current dataset, SNP-related probes, multihit probes, the cross-reactive probes identified by Chen et al. [37], and probes located on the X and Y chromosomes were discarded) and normalize that data. Low-quality probe signal sites were removed. Principal component analysis was applied to remove outliers. The Limma package [38] was used to log2-transform and normalized the gene expression data. Samples without survival time information were excluded.

## DNA differential methylation analysis

In the HCC dataset (referred to as the LIHC dataset) downloaded from the TCGA, 59 patients had matching cancer tissues and paracancerous tissues. After filtering the data and setting the 59 pairs of matching cancer and paracancerous tissues as the discovery set, the differentially methylated sites were identified. The difference between the methylation levels of tumor samples and normal tissues was calculated as β_tumor_ − β_normal_. Student’s t test was applied to compares the difference between the two groups. If the absolute value of the β difference level of a differential methylation point (DMP)’s (|β_tumor_ − β_normal_|) > 0.4 and the *p* value < 0.001, it was considered meaningful. As a high methylation level is commonly associated with inappropriate transcriptional silencing, DMPs with a β difference > 0.4 and a *p* value < 0.05 were used for further research. The same method was applied to assess the methylation data for 17 other types of cancer.

### Results

## Results of the differential methylation analysis

**Fig. 1:**
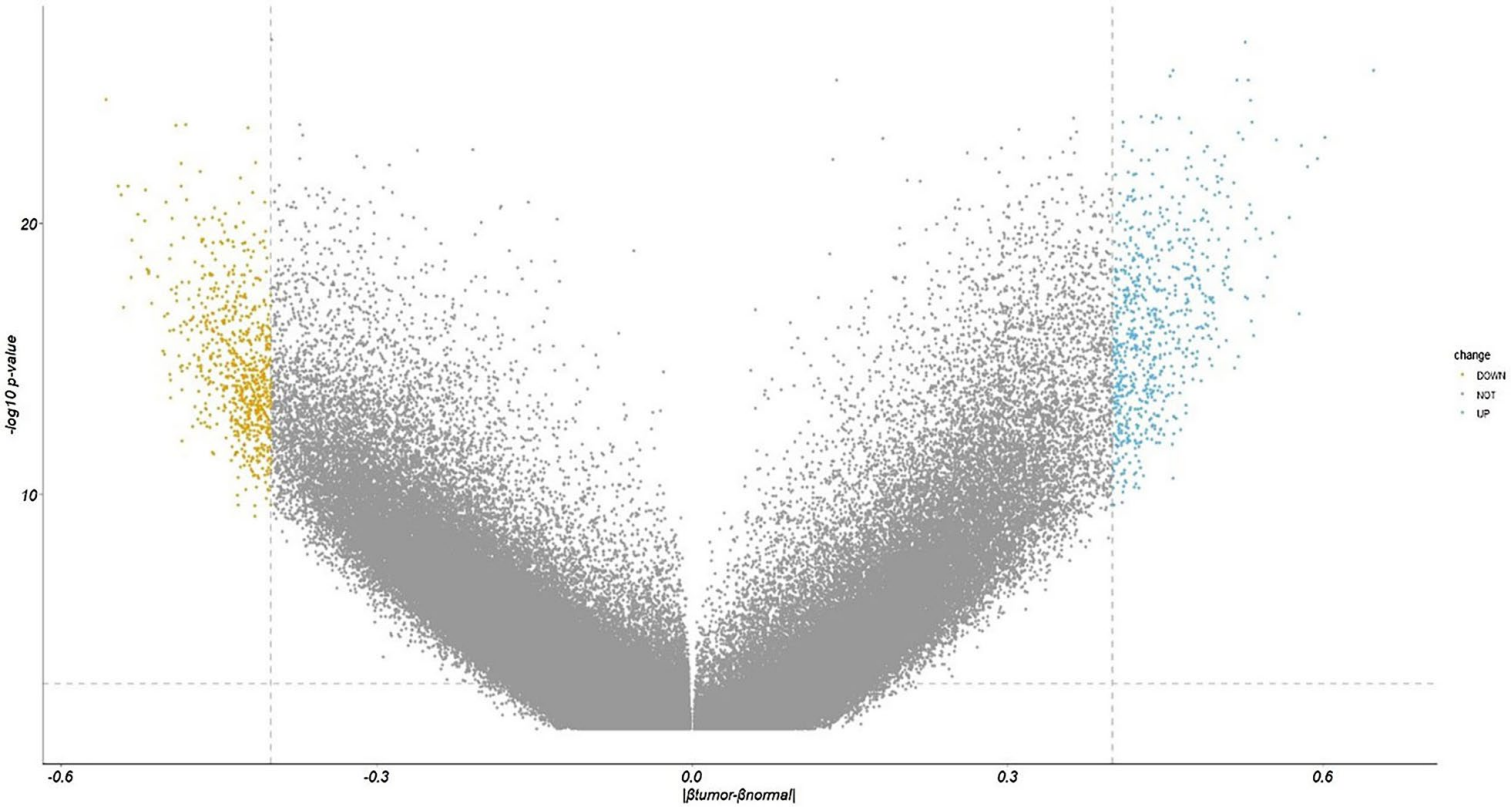
Volcano plot of the difference in methylation levels between HCC tissues and paracancerous tissues

**Fig. 2:**
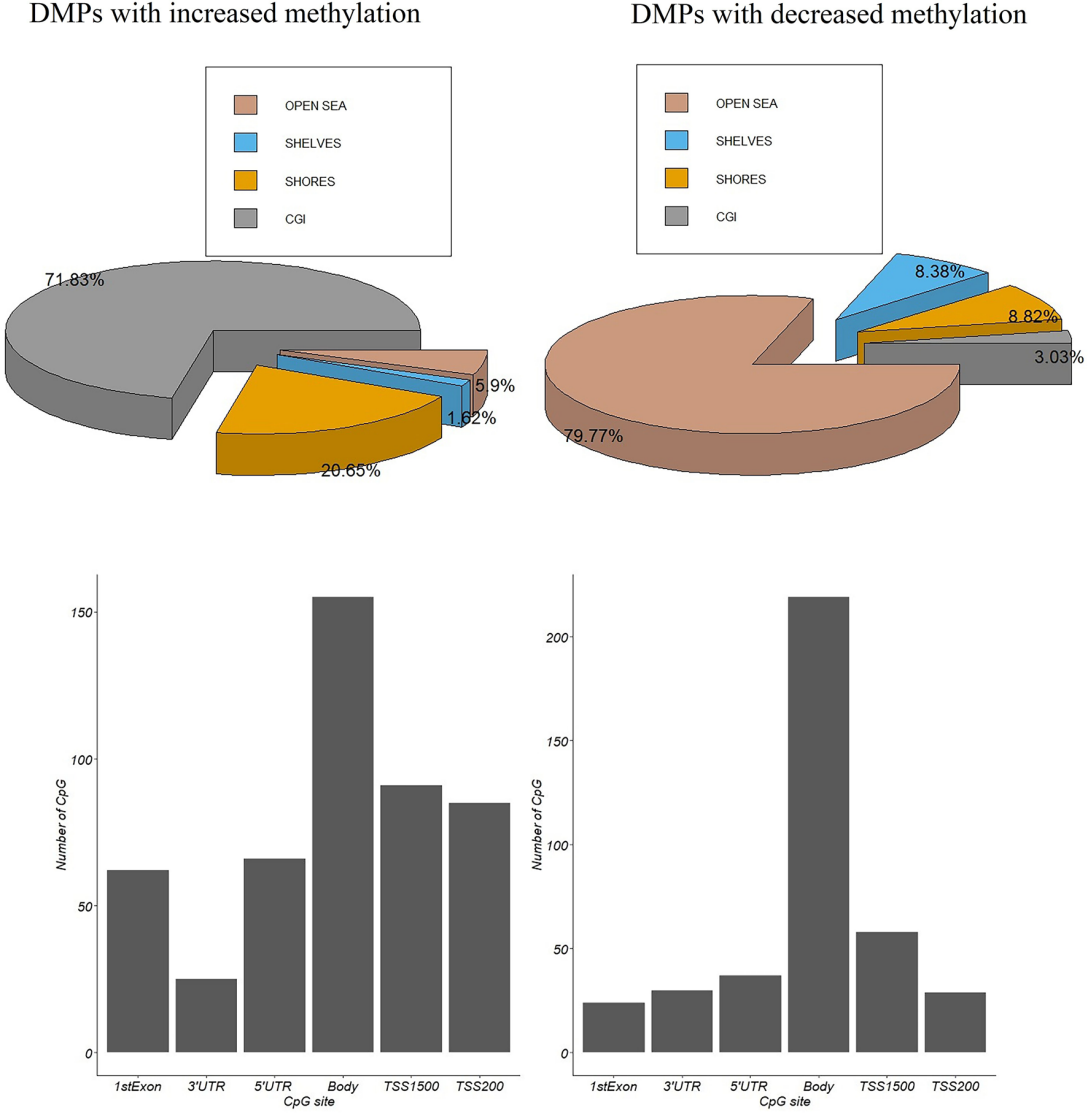
Distribution of DMPs with increased methylation and DMPs with decreased methylation in the gene

In the discovery set of the LIHC dataset (59 pairs of cancer tissues and paracancerous tissues), based on the selection standard of |β_tumor_ − β_normal_|> 0.4 and *p* value < 0.001, a total of 1374 DMPs were selected. A total of 678 DMPs had increased methylation, while 696 DMPs had decreased methylation (Fig. 1). It should be specified that the CpG distribution of different array platforms is different and that different individuals have differences in their methylation patterns [39]. Therefore, the CpG distribution displayed in this research is referenced from the Illumina HumanMethylation450 BeadChip dataset downloaded from GEO (https://www.ncbi.nlm.nih.gov/geo/query/acc.cgi?acc=gpl13534). The DMPs of the 17 other cancer types can be found in Additional file 1 (Figure S0). Among the DMPs with decreased methylation (Fig. 2), 3.03% were located within the CpG island region, and 21.9% were located within the promoter region. Moreover, 71.8% of the DMPs with increased methylation (Fig. 2) were located within the CpG island region, and 36.4% were located within the promoter region. It is worth noting that the Infinium HumanMethylation450 BeadChip array includes probes designed to target CpG islands, as well as shores, shelves and “open sea” regions. The results shown in Fig. 2 are strongly influenced by the CpGs localization and by the array design. This means that is predictable to find highly methylated CpG in the "Island" and less methylated CpGs in "open sea" [40]. In the promoter region, 69.3% of the CpG sites are located within the CpG island, meaning that high levels of gene methylation tend to occur in the promoter region, especially within the CpG islands. High methylation levels in gene promoter regions are usually the cause of transcriptional silencing, especially the silencing of cancer suppressor genes.

## Selecting the diagnostic HCC-specific sites

**Fig. 3:**
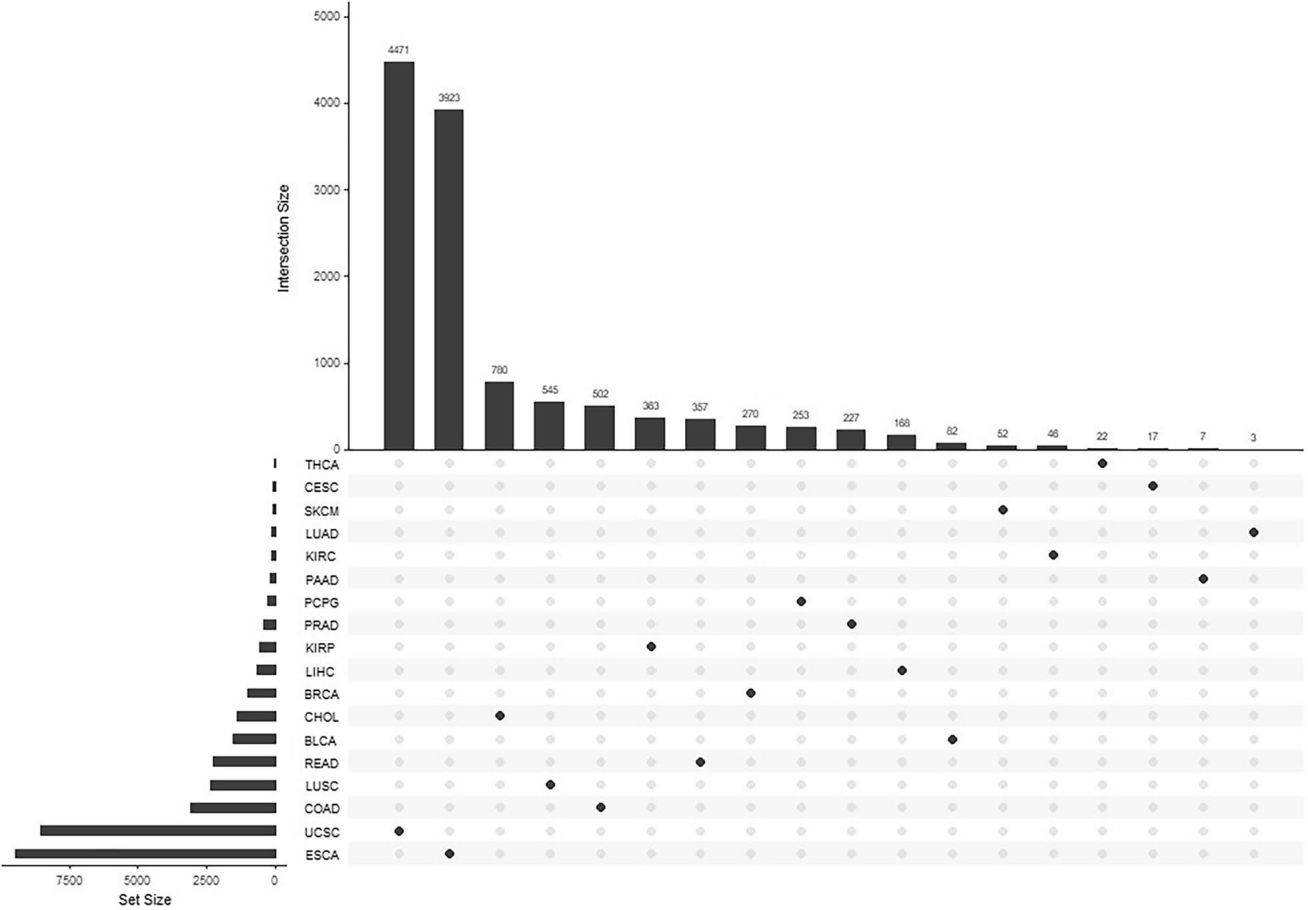
The intersection between DMPs in the LIHC dataset and 17 other datasets

From the LIHC methylation data of the discovery set, 678 DMPs with increased methylation were selected. The same standard was applied to the 17 other cancer datasets. BLCA yielded 1525 DMPs with increased methylation, BRCA yielded 1006 DMPs with increased methylation, CESC yielded 62 DMPs with increased methylation, CHOL yielded 1396 DMPs with increased methylation, COAD yielded 3074 DMPs with increased methylation, ESCA yielded 9465 DMPs with increased methylation, KIRC yielded 146 DMPs with increased methylation, KIRP yielded 577 DMPs with increased methylation, LUAD yielded 125 DMPs with increased methylation, LUSC yielded 2337 DMPs with increased methylation, PAAD yielded 157 DMPs with increased methylation, PCPG yielded 295 DMPs with increased methylation, PRAD yielded 439 DMPs with increased methylation, READ yielded 2278 DMPs with increased methylation, SKCM yielded 70 DMPs with increased methylation, THCA yielded 26 DMPs with increased methylation, and UCEC yielded 8527 DMPs with increased methylation. The UpSetR package was used to find the intersection of the 678 DMPs with increased methylation in the LIHC dataset and those for the other 17 types of cancer, as demonstrated in Fig. 3. We removed intersecting DMPs and 168 CpG sites that were specific to HCC. Of the remaining DMPs, 176 with increased methylation were in the promoter region (ranging from transcription start site (TSS200 to TSS1500). The Pearson coefficient was calculated to assess the correlation between the methylation of these 176 CpG sites and their corresponding gene expression values. If a single CpG site corresponded to multiple genes, the first one served as a reference. Sites that had a Pearson coefficient < -0.2 and a *p* value < 0.05 were considered significantly related. There were a total of 47 sites significantly related to gene expression. When these sites were overlapped with the 168 CpG sites specific to HCC, we identified 20 HCC-specific CpG sites that are in the promoter region and are significantly and negatively related to the expression of their corresponding genes.

**Fig. 4:**
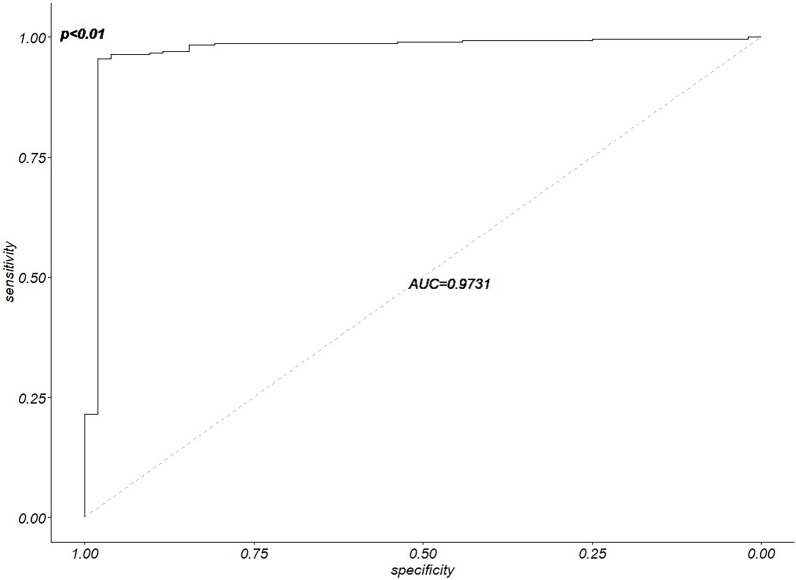
ROC curve analysis of the model

To search for relevant CpG sites related to HCC, we downloaded clinical information for 377 HCC patients. The methylation data from the 20 CpG sites and the status information of the patients were assessed by logistic regression analysis model. According to the stepwise regression method, sites with *p* values lower than 0.05 were selected. Six CpG sites matched this criterion, indicating that they can be used as diagnostic HCC-specific CpG sites (Table 1, Additional file 1: Figures S1 and S2). The relationship between the 6 CpG sites and the expression levels of their corresponding genes can be seen in the Additional file 1 (Additional file 1: Figure S3). We used a receiver operating characteristic (ROC) curve to evaluate the model, and the area under the ROC curve (AUC) was 0.97. The six diagnostic HCC-specific CpG sites had a relatively high accuracy (Fig. 4).

**Table 1:** Detailed information on HCC-specific CpG sites

ID	Gene Symbol	HR	Coefficient	95% CI for the Coefficient	*p* value
cg26581504	BCO2	3507.98	8.162797	5.40 to 11.67	2.31 × 10^–7^
cg05106294	DKK3	689.79	6.536393	2.76 to11.30	2.42 × 10^–3^
cg20342184	GRHL2	60.14	4.096723	0.29 to 8.81	5.59 × 10^–2^
cg23623667	KCNQ1	149.58	5.00	1.03 to 9.79	2.22 × 10^–2^
cg14250130	PFKP	0.001	− 6.51	(− 11.20) to (− 2.15)	3.87 × 10^–3^
cg13564825	PPP1R14A	141.75	4.95	1.64 to 8.99	7.09 × 10^–3^

## Accuracy evaluation of the model with the GEO dataset

**Fig. 5:**
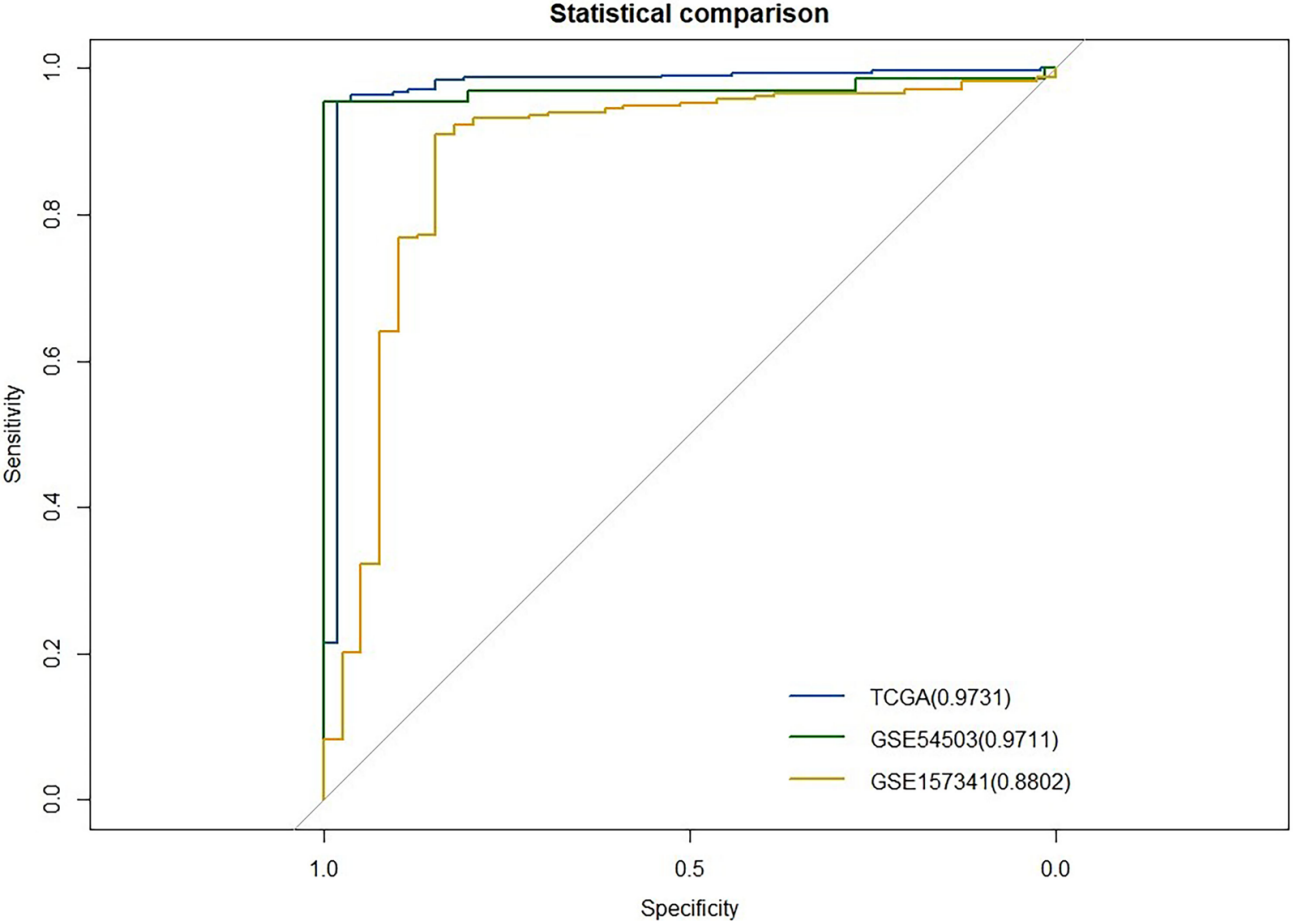
Comparison of AUC values of the model in three datasets

To further evaluate the accuracy of the six CpG sites in the TCGA dataset, we downloaded two other datasets from the GEO database that are also based on the Illumina HumanMethylation450 BeadChip (GSE54503 and GSE157341). The GSE54503 dataset includes information on 66 pairs of HCC tissues and corresponding paracancerous tissues. The GSE157341 dataset includes information on 239 cancer tissues and 35 normal liver tissues. We applied logistic regression models in the GSE54503 dataset to predict the accuracy of the six-CpG-based signature; it had relatively high accuracy within the dataset with an AUC of 0.9711. In the GSE157341 dataset, the six-CpG-based signature yielded an AUC of 0.8802. The comparisons performed with the three datasets indicate that the six-CpG-based signature has high utility (Fig. 5). Therefore, the six-CpG-based signature shows great potential in differentiating normal and cancerous tissue.

## Selecting the prognostic HCC-specific sites

We screened information in the LIHC dataset downloaded from the TCGA to find sites that are specific to HCC, are in the promoter region (ranging from TSS200 to TSS1500) and are negatively correlated with the expression of their corresponding genes. The Champ package was used to filter and normalize the data of 377 samples; 70 samples were filtered out. The remaining 307 samples were randomly divided into two sets: the training set (155 samples) and the validation set (152 samples). Finally, the entire dataset was used as another validation set.

In the training set, the methylation data were combined with survival status and survival time data. A univariate Cox regression model was constructed to assess the correlation of 47 CpG sites with the overall survival status. Finally, three CpG sites closely related to patient survival were chosen: cg08167706, cg03757145 and cg09626894 (refer to Table 2 for detailed information). The methylation level of the three sites and the coefficient from the Cox regression model were linearly combined. The formula for patients’ prognosis risk score is as follows: cg08167706 × −0.935 + cg03757145 × 1.300 + cg09626894 × 1.082. Generally, the higher the risk score is, the worse the prognosis outcome is. The relationship between the three CpG sites and the expression of their corresponding genes can be seen in Additional file 1: Figure S4.

**Table 2:** CpG sites related to patient survival

ID	Gene symbol	HR	Coefficient	95% CI for the HR	*p* value
cg08167706	AKR1B1	0.392	− 0.935	0.156–0.981	0.045
cg03757145	CDKL2	3.672	1.300	1.051–12.832	0.041
cg09626894	CFTR	2.952	1.082	1.051–8.291	0.039

## CpG sites and patient prognosis

**Fig. 6:**
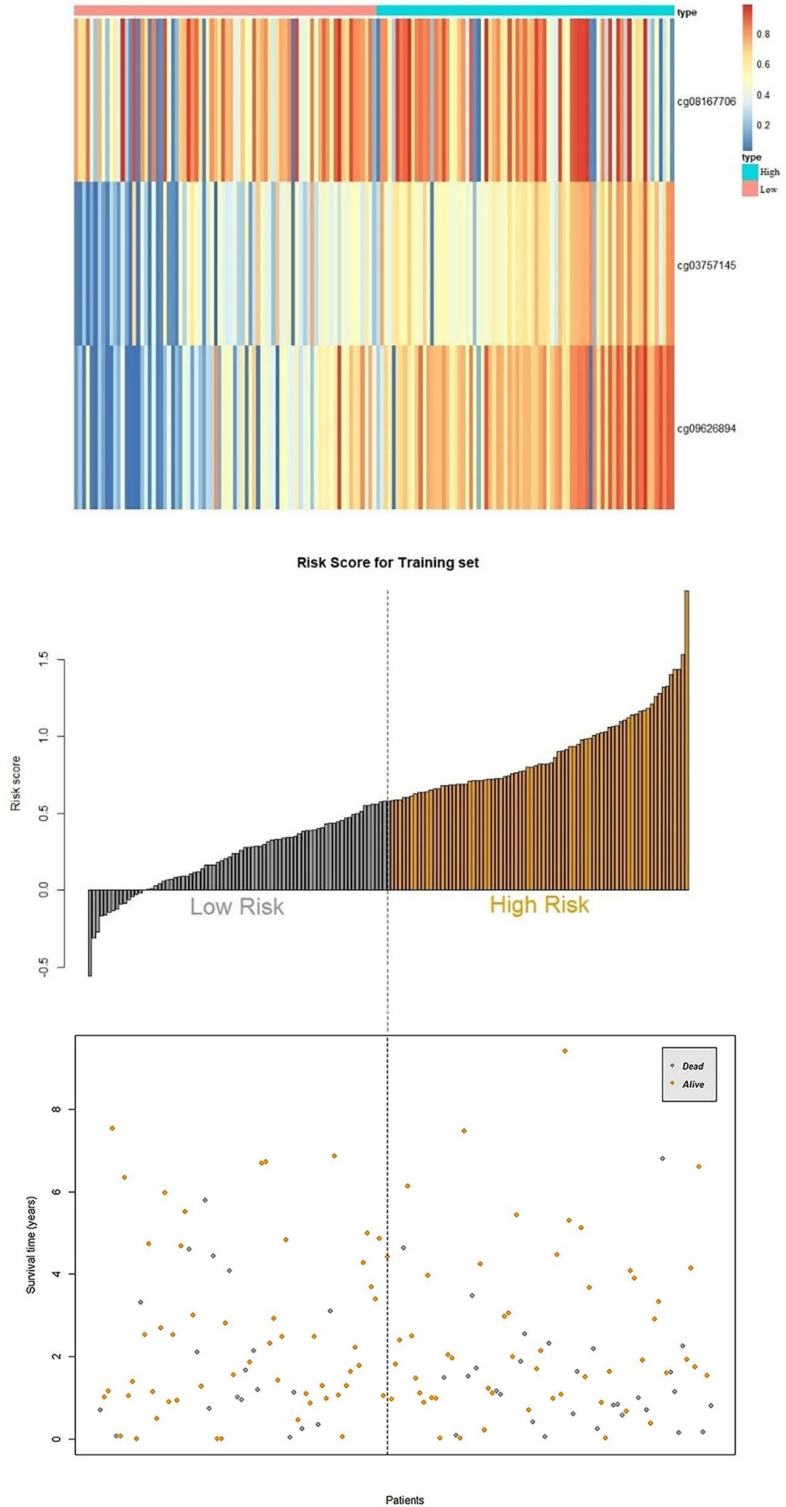
Methylation level, prognosis score and survival status for the three CpG sites

**Fig. 7:**
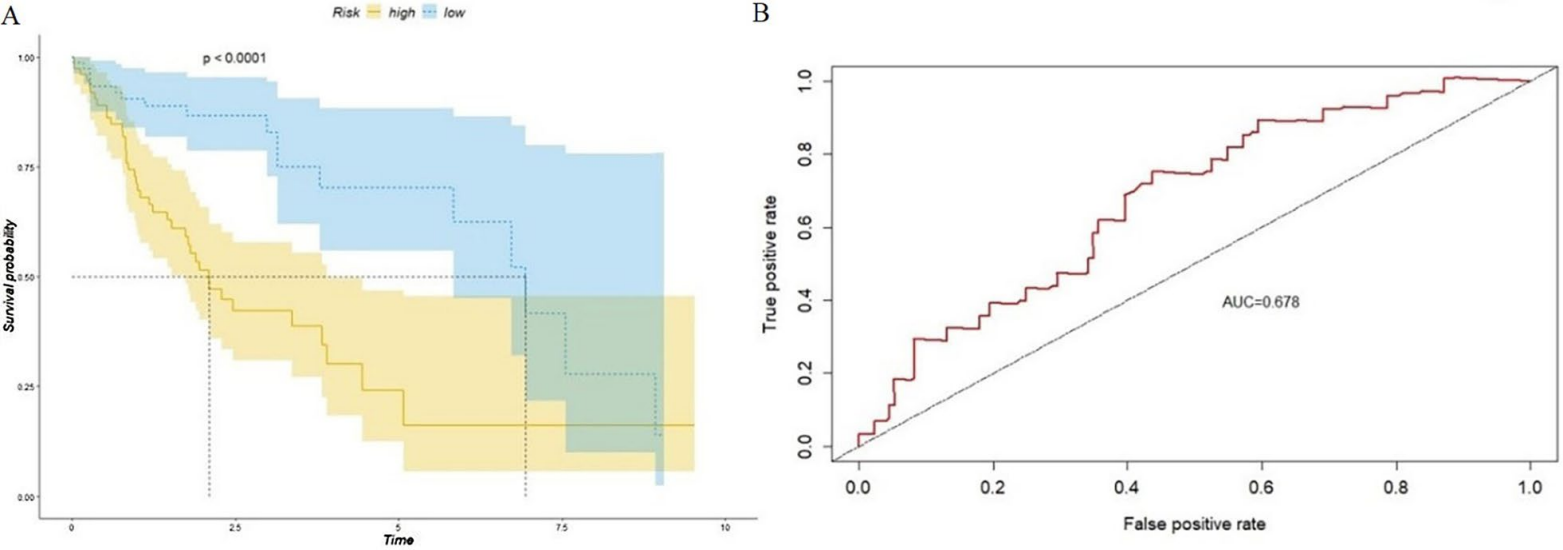
**a** Kaplan–Meier curve of the survival of the high-risk patient group and low-risk patient group in the training set and **b** ROC analysis of the prognosis scoring model

The median of scores for the three CpG sites for each of the patients (0.582) was set as the threshold to separate the patients into high-risk (n = 77) and low-risk (n = 78) groups. The prognosis scores and survival status data are shown in Fig. 6. The death rate in the high-risk group was higher than that in the low-risk group. Kaplan–Meier survival analysis and the sum of log-rank tests were used to examine and compare the difference in survival between the groups. The Kaplan–Meier survival curve (Fig. 7a) showed that the overall survival rate of the high-risk group was significantly lower than that of the low-risk group (*p* < 0.0001). The three-year survival rate and five-year survival rate for the high-risk group were 38% and 16.1%, respectively, while the three-year survival rate and five-year survival rate for the low-risk group were 78.9% and 62.4%, respectively. We applied a ROC curve to evaluate the three-CpG-based signature, and the AUC was 0.678 (Fig. 7b). This means the model has good performance. When conducting a univariate Cox regression model with the three-CpG-based signature risk score and factors such as age, sex, alcoholism, tumor grade, recurrence, clinical stage and HBV infection status, the three-CpG-based signature risk score was significantly related to the survival of patients (hazard ratio [HR] = 4.286, *p* = 0.00002, see Additional file 1: Table S1).

## Validation of the three-CpG-based signature based on the validation set and the entire dataset

**Fig. 8:**
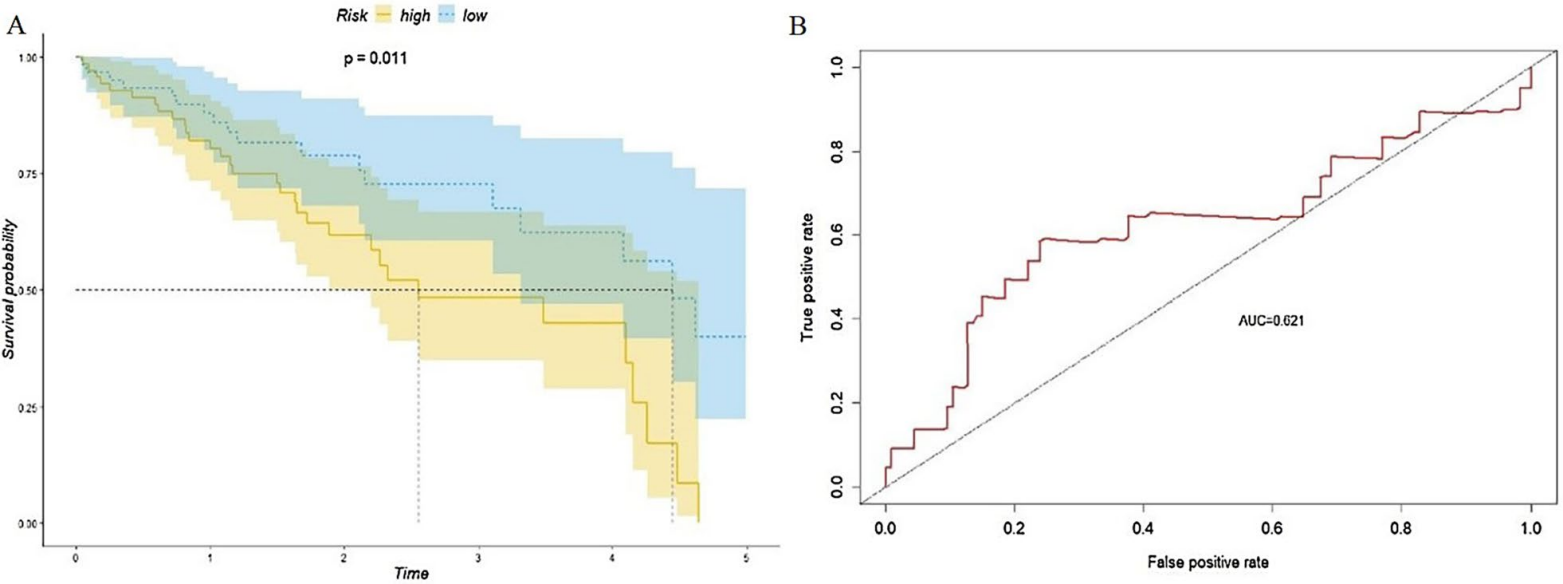
**a** Kaplan–Meier curve of the survival of the high-risk patient group and low-risk patient group in the validation set and **b** ROC analysis of the prognosis scoring model

**Fig. 9:**
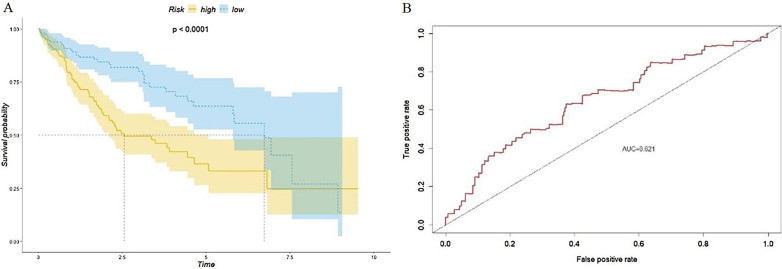
**a** Kaplan–Meier curve of the survival of the high-risk patient group and low-risk patient group in the entire dataset and **b** ROC analysis of the prognosis scoring model

To validate the utility of the prognosis model, the formula cg08167706 × -0.935 + cg03757145 × 1.300 + cg09626894 × 1.082 was applied to evaluate the prognosis of all patients. The threshold of 0.582 was used to evaluate the three-CpG-based signature. Similar to the previous approach, the patients were separated into two groups: a high-risk group (n = 80) and a low-risk group (n = 72). Kaplan–Meier survival analysis was used to compare the difference in survival of the two patient groups, and the results were similar to those of the training set analysis. The overall survival rate of the high-risk group patients was significantly lower than that of the low-risk group patients (Fig. 8a). The AUC was 0.621 (Fig. 8b). Univariate Cox regression analysis of clinical information and the three CpG sites revealed that the three-CpG-based signature was significantly related to the survival of patients (HR = 2.293, *p* = 0.026, refer to Additional file 1: Table S1). We extended the validation to the entire dataset (n = 307) and separated the patients into a high-risk patient group (n = 157) and a low-risk patient group (n = 150), and the overall survival rate of the high-risk group patients was significantly lower than that of the low-risk group patients (Fig. 9a). The AUC was 0.65 (Fig. 9b). The univariate Cox regression model indicated that the three-CpG-based signature was significantly related to patient survival (HR = 2.532, *p* = 0.0001, Additional file 1: Table S1).

## Independent predictive effect of the three-CpG-based signature

**Fig. 10:**
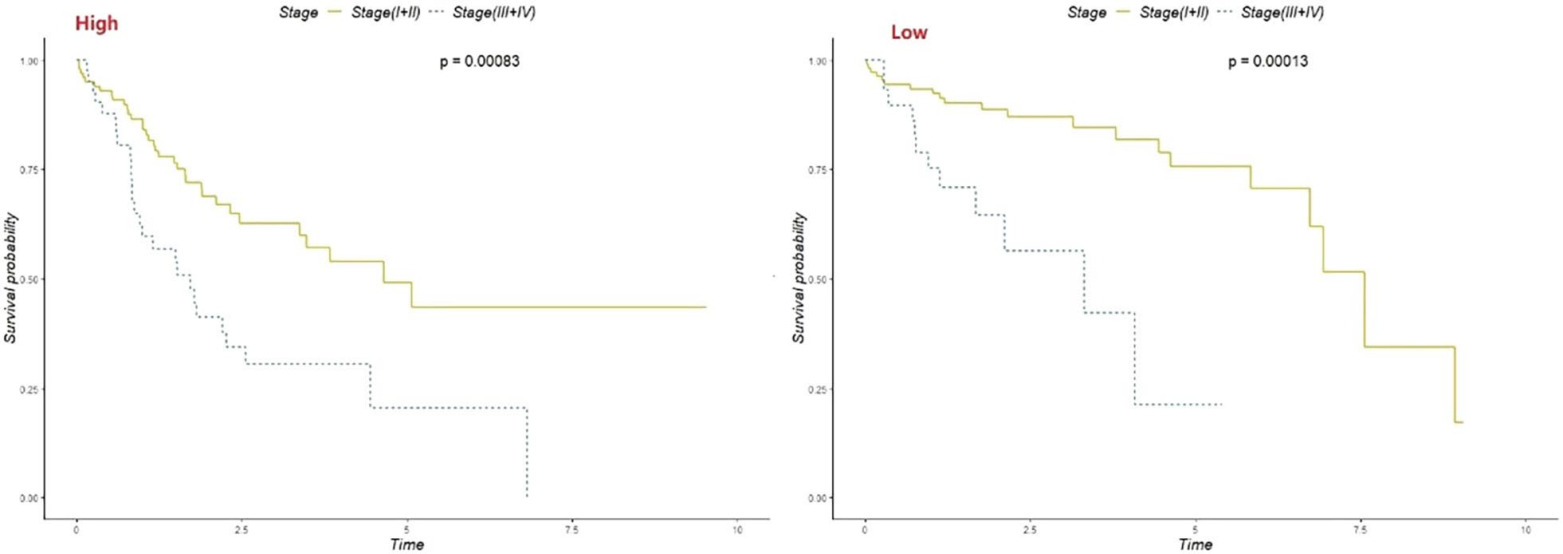
Layered analysis of clinical stage and the three-CpG-based prognosis score

To examine whether the three-CpG-based signature can predict survival independent of clinical factors, such as age, sex, alcoholism, tumor grade, recurrence, clinical stage and HBV infection, in the training set, validation set, and entire dataset, these variables were incorporated into a multivariate Cox regression model with the three-CpG-based signature. The results suggested that after adjusting for clinical factors, the three-CpG-based signature was an independent factor in the training set (HR = 3.971, *p* < 0.01), the validation set (HR = 2.405, *p* < 0.01) and the entire dataset (HR = 2.205, *p* < 0.01). In addition, it was discovered that the clinical stage was also significantly related to the survival of patients. In the layered analysis for the three-CpG-based signature and clinical staging, the patients were separated into a high-risk group and a low-risk group. Regardless of whether the patient belonged to the high-risk group or the low-risk group, the survival rate for patients who were in stage III and stage IV was significantly lower than that for those in stages I and II (Fig. 10, *p* < 0.01). Finally, a nomogram was developed to evaluate the influence of each of the variables on prognosis (Additional file 1: Figure S5). As shown in the figure, the three-CpG-based signature contributed the most to the prognosis. This means that the three-CpG-based signature was the best indicator of patient survival. Finally, the flowchart of the search process for CpG sites used for diagnosis and prognosis is shown in the Additional file 1: Figure S6.

### Discussion

Gene methylation is a symbol of tumor occurrence. The occurrence of every type of tumor is related to high gene methylation levels. To identify biomarkers that are specific to HCC and can aid in its diagnosis, methylation data for HCC and 17 other types of cancer based on the Illumina 450 K DNA array were downloaded from the TCGA. Based on the criteria of absolute β difference > 0.4 and *p* value < 0.001, we filtered out DMPs with increased methylation that were not specific to HCC. We applied logistic regression analysis and identified 6 CpG sites that are unique to HCC and can be used to diagnose HCC: cg26581504 (BCO2), cg05106294 (DKK3), cg20342184 (GRHL2), cg23623667 (KCNQ1), cg14250130 (PFKP), and cg13564825 (PPP1R14A). These CpG sites can be used to effectively distinguish between cancer tissues and normal liver tissues. The AUC for the ROC curve of the model incorporating these sites is 0.973. We also used two independent HCC datasets from the GEO to validate the accuracy of these biomarkers. The AUCs for the ROC curves were 0.971 and 0.880. This means that these 6 biomarkers have high utility for the diagnosis of HCC. What separates this research from a previous study on diagnostic markers is that this study is the first to use the intersection method to filter highly altered DMPs between different TCGA datasets. The results are different between the two studies [33, 34].

In the diagnosis of HCC, this six-CpG-based signature has relatively high sensitivity and specificity. For patients who are already diagnosed with HCC, we also hope to develop a CpG-based signature that can evaluate the prognosis of patients for early intervention and disease management. Due to the lack of survival information and clinical data that can be used for validation, this research separated samples in the LIHC dataset from TCGA into two sets: a training set and a validation set. Analysis of the training set revealed three CpG sites as candidate biomarkers related to prognosis: cg08167706 (AKR1B1), cg03757145 (CDKL2) and cg09626894 (CFTR). These three sites were used as the basis for prognosis scoring. According to the prognosis scores, the patients were divided into a high-risk group and a low-risk group, and the overall survival rate of the high-risk group was significantly lower than that of the low-risk group (*p* < 0.001). The model was then validated with the validation set and the whole dataset, and ROC analysis was performed. The results showed that the three-CpG-based prognosis evaluation model can be used to effectively evaluate the prognosis of patients. Furthermore, clinical variables and the three-CpG-based signature were put into univariate and multivariate Cox regression models. Nomograms were also constructed for these clinical variables and the three-CpG-based signature. These results indicate that the signature is an important independent factor for predicting patient survival.

### Conclusions

In summary, this study identified a six-CpG-based signature that may be used for diagnosing HCC and a three-CpG-based signature for predicting the survival of patients with HCC through analyzing the methylation profile and clinical data from TCGA and GEO.

In the diagnosis of HCC, taking tissues for methylation sequencing is certainly invasive. Plasma ctDNA assessment is both minimally invasive and can provide more dynamic monitoring of cancer. Clinically, peripheral plasma ctDNA is already applied for the early screening and detection of cancer. In HCC, the methylation levels of tumor DNA and matched plasma ctDNA are highly correlated [34]. Research indicates that CpG assessment of HCC tissue samples is no more effective than that of ctDNA samples [41]. In future research, we will gather our own samples and apply the six-CpG-based signature and the three-CpG-based signature for the assessment of HCC tissues and peripheral plasma ctDNA to find the best diagnosis and prognosis plan for HCC patients.

## Supplementary Information

**Additional file 1**. **Table S1.** Univariate and multivariate Cox regression analysis for the whole dataset. **Figure S0.** Volcano plot of the difference inmethylation levels for the cancer tissues and paracancerous tissues of 17 other cancer types. **Figure S1.** Methylation levels of the six HCC-specific CpG sites. **Figure S2.** Coefficients for the six HCC-specific CpG sites based on the univariate Cox regression model. **Figure S3.** Six HCC-specific CpG sites used for diagnosis and the expression levels of their corresponding genes. **Figure S4.** Three CpG sites related to patient survival and the expression levels of their corresponding genes. **Figure S5.** Nomogram of clinical factors and the three-CpG-based prognosis score. **Figure S6.** The flowchart of searching process of CpG sites used for diagnosis and prognosis

## Data Availability

TCGA: https://tcga-data.nci.nih.gov, now hosted at GDC: https:// portal.gdc.cancer.gov/. GSE157341: https://www.ncbi.nlm.nih.gov/geo/query/acc.cgi?acc=GSE157341. GSE54503: https://www.ncbi.nlm.nih.gov/geo/query/acc.cgi?acc=GSE54503

## Abbreviations

LIHC: Liver hepatocellular carcinoma
TCGA: The Cancer Genome Atlas
GEO: Gene Expression Omnibus
DNMTs: DNA methyltransferases
MGMT: O^6^-methylguanine-DNA-methyltransferase
DMP: Differential methylation point
TSS: Transcription start site
