# Supplementary material for: Screening the genome for HCC-specific CpG methylation signatures as biomarkers for diagnosis and prognosis evaluation
Source: BMC Med Genomics. 2021 Jun 19;14:163. doi: 10.1186/s12920-021-01015-9 (PMC8214801; doi:10.1186/s12920-021-01015-9)
Supplement: Supplementary file 1 — Additional file 1. Table S1. Univariate and multivariate Cox regression analysis for the whole dataset. Figure S0. Volcano plot of the difference inmethylation levels for the cancer tissues and paracancerous tissues of 17 other cancer types. Figure S1. Methylation levels of the six HCC-specific CpG sites. Figure S2. Coefficients for the six HCC-specific CpG sites based on the univariate Cox regression model. Figure S3. Six HCC-specific CpG sites used for diagnosis and the expression levels of their corresponding genes. Figure S4. Three CpG sites related to patient survival and the expression levels of their corresponding genes. Figure S5. Nomogram of clinical factors and the three-CpG-based prognosis score. Figure S6. The flowchart of searching process of CpG sites used for diagnosis and prognosis [file 12920_2021_1015_MOESM1_ESM.docx]

**Table S1** Univariate and multivariate Cox regression analysis for the whole dataset

| Variables | Univariable Analysis | | | Multivariable Analysis | | |
| --- | --- | --- | --- | --- | --- | --- |
|  | HR | 95% CI for the HR | P-value | HR | 95% CI for the HR | P-value |
| **Training Set (n=155)** |  |  |  |  |  |  |
| Age | 1.004 | 0.982-1.027 | 0.702 | 1.001 | 0.978-1.03 | 0.920 |
| Sex (Female) | 1.059 | 0.562-1.996 | 0.856 | 0.944 | 0.465-1.92 | 0.872 |
| Alcohol (No) | 1.092 | 0.584-2.039 | 0.781 | 1.097 | 0.547-2.20 | 0.795 |
| Grade (1+2) | 0.771 | 0.417-1.426 | 0.408 | 0.576 | 0.298-1.11 | 0.101 |
| Recurrence (No) | 1.453 | 0.796-2.651 | 0.222 | 1.024 | 0.528-1.99 | 0.943 |
| Stage (I+II) | 2.937 | 1.559-5.531 | 0.0008 | 2.113 | 1.062-4.20 | 0.033 |
| HBV (Negative) | 1.764 | 0.864-3.599 | 0.118 | 1.390 | 0.645-3.00 | 0.400 |
| Risk Score | 4.286 | 2.167-8.476 | 0.00002 | 3.971 | 1.923-8.20 | 0.000194 |
| **Validation Set (n=152)** |  |  |  |  |  |  |
| Age | 1.002 | 0.981-1.023 | 0.811 | 1.013 | 0.992-1.03 | 0.225 |
| Sex (Female) | 0.855 | 0.474-1.541 | 0.602 | 1.454 | 0.732-2.89 | 0.284 |
| Alcohol (No) | 0.771 | 0.398-1.491 | 0.440 | 0.716 | 0.345-1.49 | 0.370 |
| Grade (1+2) | 1.108 | 0.617-1.988 | 0.729 | 0.852 | 0.461-1.58 | 0.6105 |
| Recurrence (No) | 2.441 | 1.300-4.581 | 0.005 | 1.900 | 0.987-3.66 | 0.054 |
| Stage (I+II) | 3.649 | 1.978-6.731 | 0.00003 | 3.572 | 1.793-7.11 | 0.0002 |
| HBV (Negative) | 0.969 | 0.521-1.804 | 0.922 | 1.200 | 0.634-2.27 | 0.575 |
| Risk Score | 2.293 | 1.104-4.763 | 0.026 | 2.405 | 1.108-5.22 | 0.027 |
| **Entire Set** |  |  |  |  |  |  |
| Age | 1.010 | 0.995-1.026 | 0.164 | 1.011 | 0.996-1.03 | 0.157 |
| Sex (Female) | 0.874 | 0.574-1.330 | 0.530 | 1.021 | 0.647-1.61 | 0.929 |
| Alcohol (No) | 0.997 | 0.636-1.562 | 0.991 | 0.880 | 0.537-1.44 | 0.613 |
| Grade (1+2) | 1.038 | 0.684-1.575 | 0.859 | 0.938 | 0.610-1.44 | 0.768 |
| Recurrence (No) | 1.558 | 1.022-2.375 | 0.038 | 1.207 | 0.779-1.87 | 0.399 |
| Stage (I+II) | 3.046 | 1.997-4.646 | 2.3×10^-7^ | 2.750 | 1.757-4.30 | 9.6×10^-6^ |
| HBV (Negative) | 1.078 | 0.699-1.662 | 0.732 | 1.254 | 0.804-1.95 | 0.318 |
| Risk Score | 2.532 | 1.577-4.065 | 0.0001 | 2.025 | 1.254-3.27 | 0.003 |





**Figure S0** Volcano plot of the difference in methylation levels for the cancer tissues and paracancerous tissues of 17 other cancer types


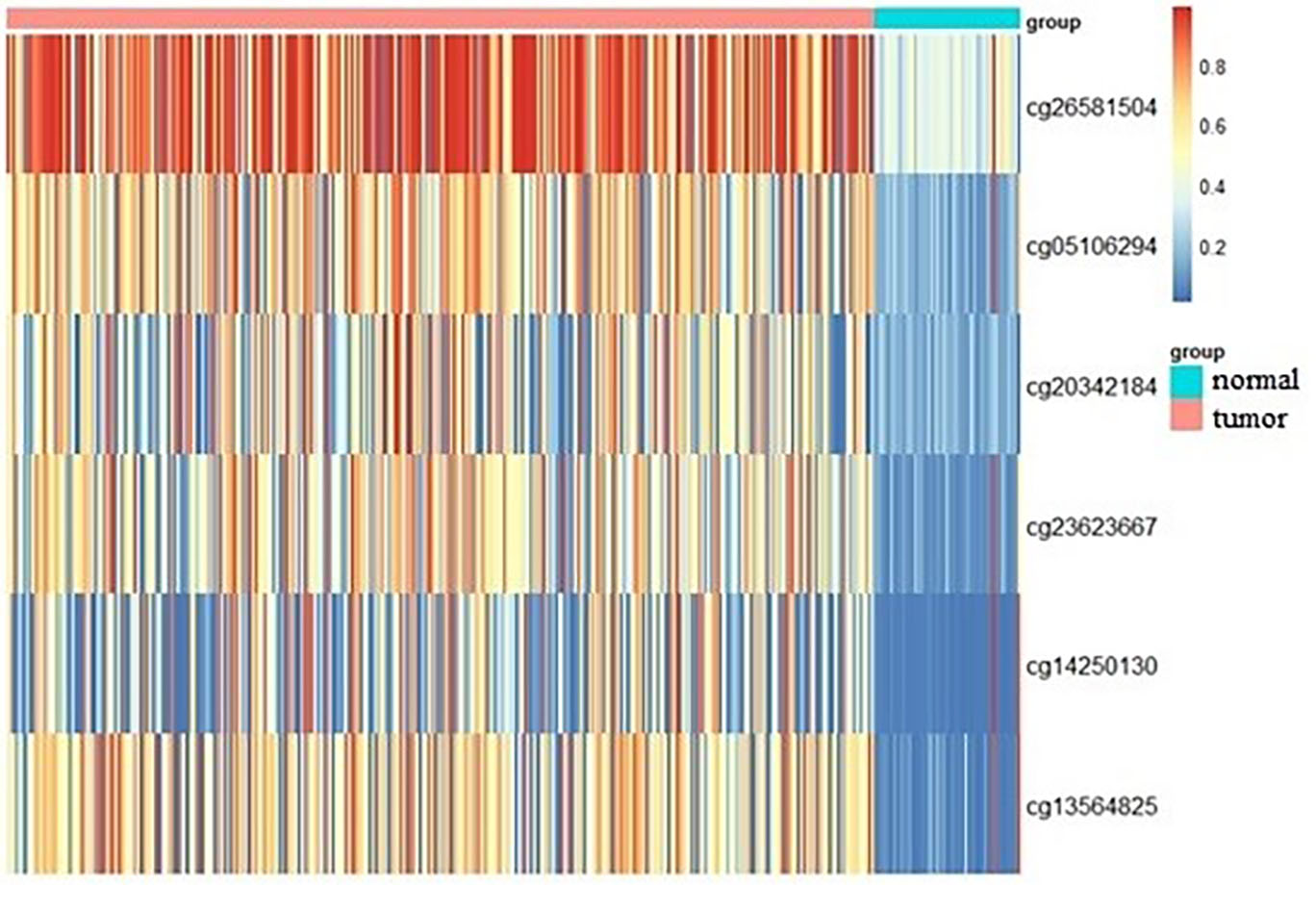


**Figure S1** Methylation levels of the six HCC-specific CpG sites


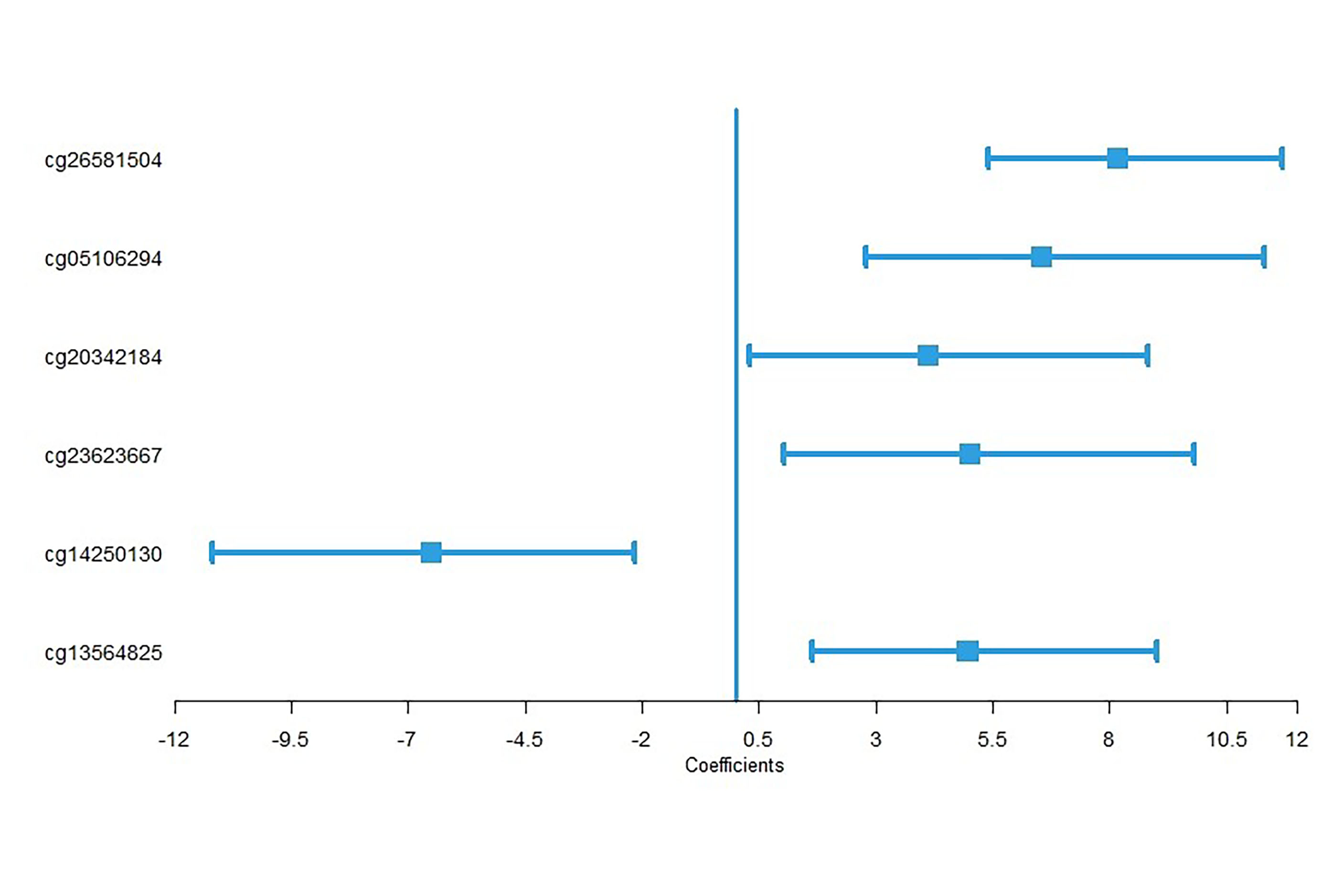


**Figure S2** Coefficients for the six HCC-specific CpG sites based on the univariate Cox regression model


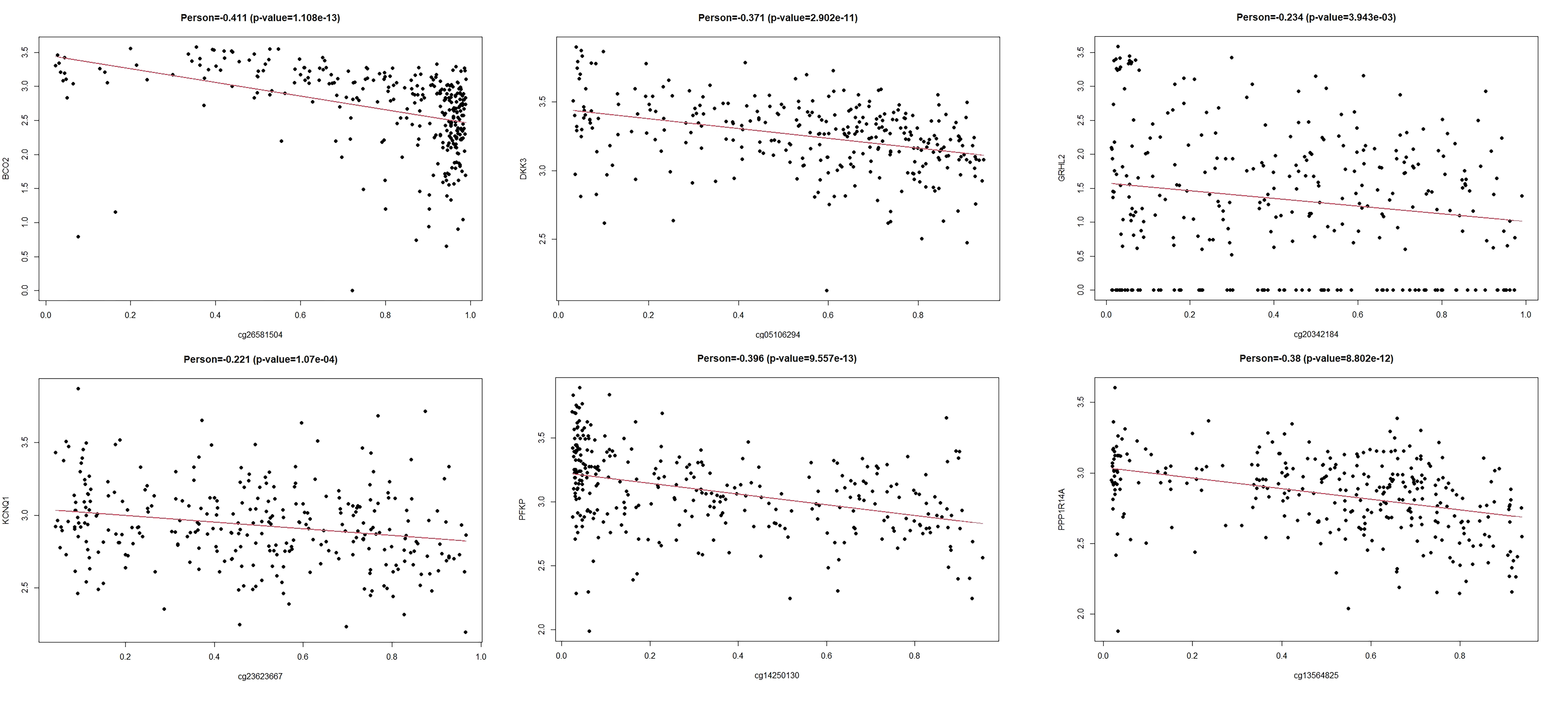


**Figure S3** Six HCC-specific CpG sites used for diagnosis and the expression levels of their corresponding genes


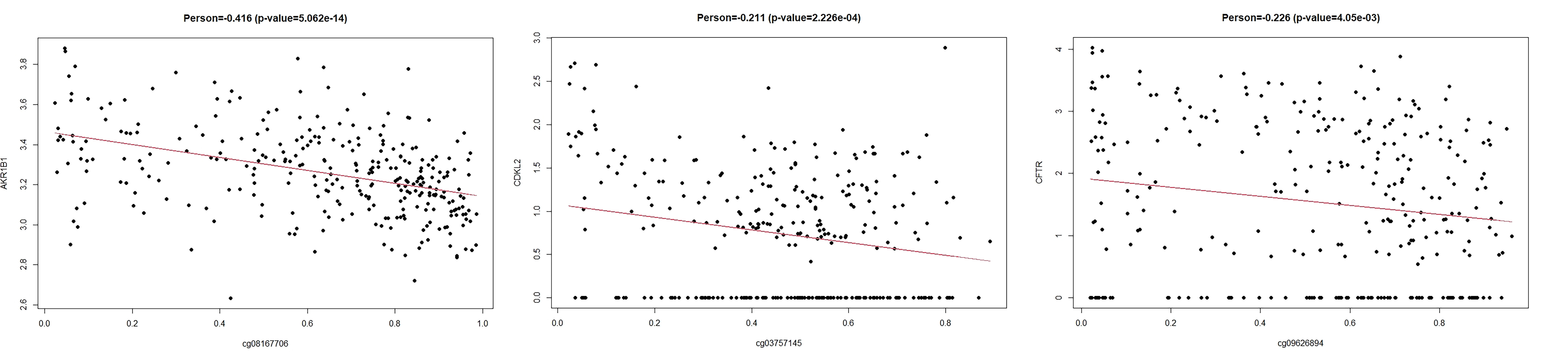


**Figure S4** Three CpG sites related to patient survival and the expression levels of their corresponding genes


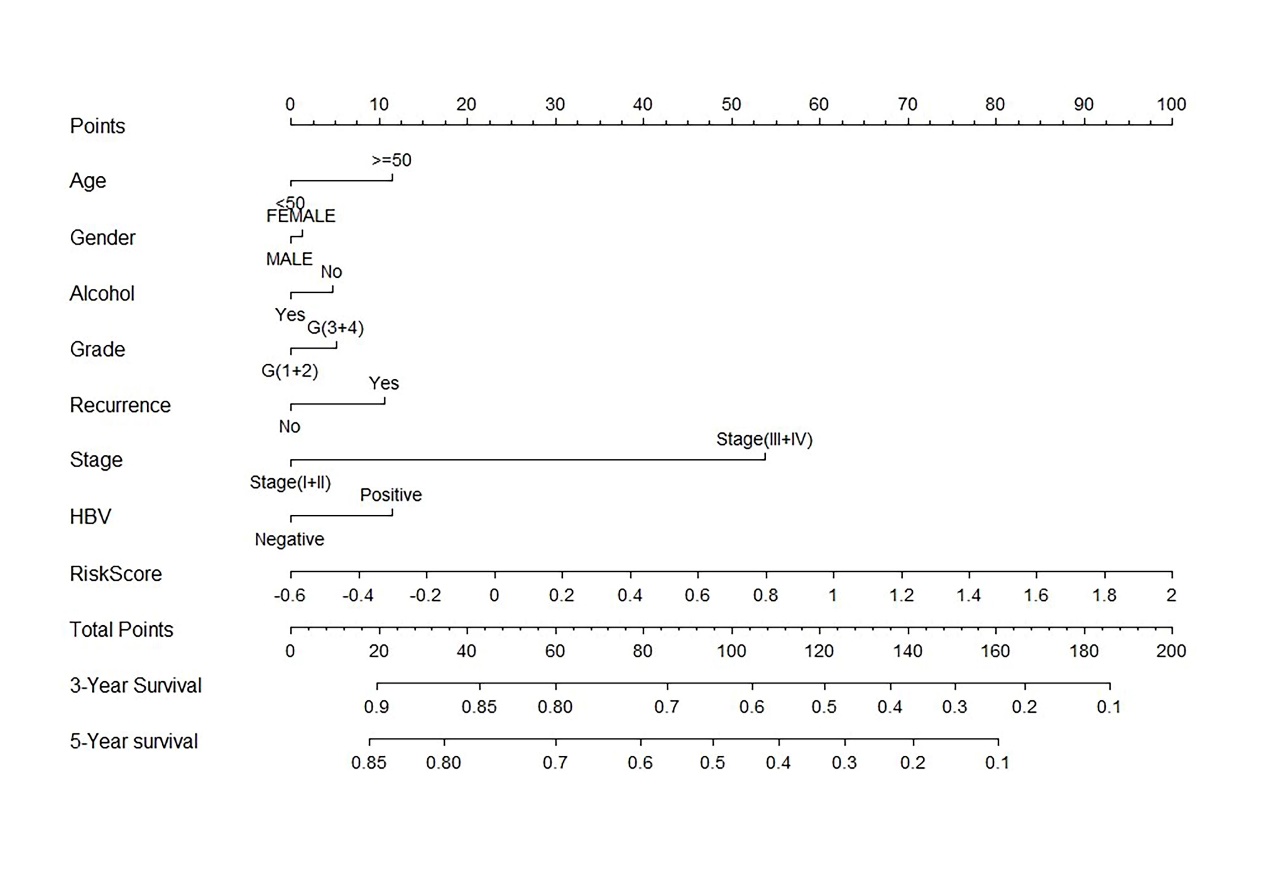


**Figure S5** Nomogram of clinical factors and the three-CpG-based prognosis score





Figure S6 the flowchart of searching process of CpG sites used for diagnosis and prognosis
